# Paeoniflorin reduces the inflammatory response of THP-1 cells by up‐regulating microRNA-124: Paeoniflorin reduces the inflammatory response of THP-1 cells through microRNA-124

**DOI:** 10.1007/s13258-021-01083-2

**Published:** 2021-03-29

**Authors:** Danyun Huang, Zhijun Li, Yue Chen, Yan Fan, Tao Yu

**Affiliations:** 1 Dermatology, The Second Affiliated Hospital, Zhejiang Chinese Medical University, Hangzhou, 310005 Zhejiang China; 2 Department of Internal Medicine, Huangyan District Hospital of Traditional Chinese Medicine, Taizhou, 318020 Zhejiang China; 3 Dermatology, Hangzhou Traditional Chinese Medicine Hospital, Dingqiao Campus, Hangzhou, 310006 Zhejiang China

**Keywords:** Paeoniflorin, THP-1 cells, miRNA-124, Proliferation, Apoptosis

## Abstract

**Background:**

The activation of macrophages and the release of inflammatory cytokines are the main reasons for the progress of systemic lupus erythematosus (SLE). MicroRNA (miRNA)-124 is involved in the regulation of macrophages and is a key regulator of inflammation and immunity.

**Objective:**

To explore whether paeoniflorin (PF) regulates the biological functions of macrophages depends on miR-124.

**Methods:**

RT-PCR, WB, ELISA, CCK-8 and flow cytometry were used to evaluate that PF regulated the biological functions of THP-1 cells through miR-124.

**Results:**

PF significantly inhibited the proliferation while promotes the apoptosis of THP-1 cells, and inhibited the release of IL-6, TNF-α and IL-1βin THP-1 cells. RT-PCR results shown that PF up-regulated the expression of miR-124 in THP-1 cells. Functional recovery experiments showed that compared with the LPS + mimic-NC group, LPS + miR-124 mimic significantly inhibited the proliferation and the release of IL-6, TNF-α and IL-1β, but promoted the apoptosis of THP-1 cells. In addition, compared with the LPS + PF + inhibitor-NC group, LPS + PF + miR-124 inhibitor significantly promoted the proliferation and the release of IL-6, TNF-α and IL-1β, but inhibited the apoptosis of THP-1 cells.

**Conclusions:**

By down-regulating miR-124, PF inhibits the proliferation and inflammation of THP-1 cells, and promotes the apoptosis of THP-1 cells.

## Introduction

Systemic lupus erythematosus (SLE) is a chronic autoimmune disease, and innate immune cells and cytokines play key roles in its pathology (Kiriakidou and Ching 2020). Studies on SLE patients and animal models have shown that the activation of macrophages and the abnormalities of related cytokines are closely related to SLE (Ma et al. 2019; Zhang et al. 2020). Macrophages are mainly divided into two subtypes, M1 (pro-inflammatory phenotype) and M2 (anti-inflammatory phenotype) (Funes et al. 2018). And induction of macrophage polarization into the M2 phenotype is helpful to reduce experimental SLE (Li et al. 2016). Therefore, the inhibition of macrophage proliferation and induction of M2 macrophage polarization are helpful to alleviate the disease progression of SLE.

Recently, studies have reported that paeoniflorin (PF), an extract of paeonia lactiflora root, inhibits the proliferation of a variety of cells, such as human mesangial cells (Liu et al. 2019), B cells (Zhang et al. 2015) and vascular smooth muscle cells (Fan et al. 2018). However, the effect of PF on the proliferation and the polarization of macrophages are not yet clear. And, we speculate that PF might exert certain clinical value in the treatment of SLE patients via regulating the proliferation and the polarization of macrophages.

MicroRNA (miRNA)-124 has become a key regulator of inflammation and immunity, and is involved in the regulation of various innate and adaptive immune cells, including hematopoietic cells, CD4^+^ T cells and macrophages (Qin et al. 2016). Among them, miR-124 inhibits the proliferation of macrophages (Zhai et al. 2020), and is essential for inducing and maintaining the M2 phenotype of macrophages (Veremeyko et al. 2013). Therefore, we speculate that PF might inhibit the proliferation and promote the polarization of macrophages by promoting the expression of miRNA-124.

TNF-α, IL-6 and IL-1β contributes to the survival of macrophages (Fan et al. 2020; Meng et al. 2010; Yao et al. 2018). Many studies have reported that miRNA-124 inhibits the expression of TNF-α, IL-6 and IL-1β (He et al. 2019; Ohnuma et al. 2019). However, whether PF inhibit the production of TNF-α, IL-6 and IL-1β by up-regulating the expression of miRNA-124 in macrophages is still unknown.

Based on the basis above, this study aims to explore whether there exists the potential mechanism of PF/miRNA-124 in macrophages, and the effects of PF/miRNA-124 on the proliferation and polarization of macrophages. These findings of this study might provide new ideas for the treatment of SLE patients.

## Materials and methods

### Cell culture

Established THP-1 human macrophages (Wang et al. 2016) were purchased from Institute of Cell Research, Chinese Academy of Sciences (Shanghai, China). THP-1 macrophages were cultured in RPMI-1640 containing 10% fetal bovine serum (from Gibco; Thermo Fisher Scientific, Inc.) and 100 U/ml penicillin. We treated THP-1 cells with 100 ng/ml phorbol 12-myristate 13-acetate (PMA, AdipoGen, USA) for 24 h to generate M0 macrophages (M0) (Chanput et al. 2013). 100 ug/ml lipopolysaccharide (LPS) was treated with THP-1 cells (1–1.5 × 10^8^) for 48 h to induced M1 macrophages. PF (MedCham Express) was dissolved in dimethyl sulfoxide (DMSO). Cells were treated with various concentrations of PF (0,1, 10, 100, 500, 1000, 2000 and 3000 ug/ml) for 48 h, and appropriate controls were treated with DMSO at the same concentrations. The cells were placed in a cell incubator containing 5 % CO_2_ at 37 °C for cultivation.

### Cell viability assays

Cell viability was measured by using a Cell Counting Kit-8 (CCK-8, Dojindo, Japan) according to the manufacturer’s instructions. Briefly, cells were seeded at a density of 0.3 × 10^4^ cells/well in 96-well plates, and incubated in 1640 medium overnight. Then, CCK-8 solution (10 ul) and 90 ul 1640 medium (serum free) was added to each well for 2 h and optical density was measured at 450 nm. The proliferation of cells was detected at 12 h, 24 h, 36 and 24 h.

### Quantitative real‐time (RT-PCR)

For RT-PCR analysis, total RNA was isolated by using the TRIzol reagent (Takara Bio, Inc), and reverse transcription was performed using the PrimeScript RT reagent kit (Takara Bio, Inc) according to manufacturer’s instructions. The primers used in this study were shown as follow: GAPDH (forward sequence (F): 5′–GAAGGTGAAGGTCGGAGTC–3′ and reverse sequence (R): 5′–GAAGATGGTGATGGGATTTC–3′), IL-6 (F: 5′–ATGAACTCCTTCTCCACAAGC–3′ and R: 5–CTACATTTGCCGAAGAGCCCTCAGGCTGGACTG–3′), TNF-α (F:5′–ATGAGCACTGAAAGCATGATC–3′ and R:5′–TCACAGGGCAATGATCCCAAAGTAGACCTGCCC–3′), IL-1β (F: 5′–ATGATGGCTTATTACAGTGGCAA–3′ and R: 5′–GTCGGAGATTCGTAGCTGGA–3′), miR-124 (5′–GCGAGGATCTGTGAATGCCAAA–3′) and U6 (CD201-1045, Provided by Tiangen Biotech). GAPDH and U6 were used as negative control. The gene expression analysis was performed by quantitative real-time PCR (StepOne Plus; Applied Biosystems, USA) with standard SYBR-Green PCR kits (Takara Bio, Inc). Reactions were conducted by an initial incubation at 95 °C for 2 min, followed by 40 cycles of 95 °C for 15 s and 60 °C for 30 s. The relative expression of each target gene normalized to GAPDH was calculated using the 2^−ΔΔct^ method.

### Western blot

We lysed the cells using a protein extraction reagent (Beyotime, Jiangsu, China) in the presence of protease and phosphatase inhibitor cocktail tablets. We measured protein concentration using a BCA Protein Assay Kit (Beyotime, Jiangsu, China). Soluble lysates containing about 50 µg proteins per sample were resolved with sodium dodecyl sulfate–polyacrylamide gel electrophoresis and transferred to a polyvinylidene fluoride membrane (Merck Millipore). After blocking using 10 % fat-free milk, membranes were incubated with primary antibodies against IL-6 (1:100, CST), TNF-α (1:100,CST), IL-1β (ab234437, abacam) and β-actin (1:100, CST) overnight at 4 °C overnight and secondary antibodies (1:1000) at room temperature for 1 h. After several washes, the immunoblot was detected with enhanced chemi-luminescence (Pierce Biotechnology) according to the manufacturer’s instructions.

### Apoptosis assay

After drug treatment for 48 h, cells were harvested and washed twice with PBS and resuspended in binding buffer, then the cells were double-stained with Annexin V-Phycoerythrin (PE) and 7-aminoactinomycin (7-AAD) (BD Biosciences) for 15 min at room temperature in the dark. Subsequently, cells were analyzed by flow cytometry using Calibur (BD Biosciences) within 1 h.

### 2.6 Cell transfection

miRNA-124 mimic and inhibitor were purchased from GenePharma (Shanghai, China). The miRNA-124 mimic and miRNA-124 inhibitor were designed according to the sequence: 5′–GCGAGGATCTGTGAATGCCAAA–3′. Inhibitor-Negative Control (inhibitor-NC) and mimic-NC were provided by GenePharma (Shanghai, China). Cells were transfected with 25–50 nM of the indicated miRNA-124 mimic, mimic-NC, miRNA-124 inhibitor and inhibitor-NC using Lipofectamine 200 reagent (GenePharma, Shanghai, China).

### ELISA

The Human TNF alpha ELISA Kit (ab181421, abacam), Human IL-6 ELISA Kit (ab178013, abacam) and Human IL-1 beta ELISA Kit (ab214025, abacam) were used to detected the expression of TNF-α, IL-6 and IL-1β in the cell lysate and cell supernatant of THP-1 cells. Each experiment was repeated at least three times.

### Bioinformatics prediction

The 2D and 3D structure of PF was provided by PubChem (https://pubchem.ncbi.nlm.nih.gov/). The potential mechanism involved in PF was predicted via the Traditional Chinese Medicine Systems Pharmacology Database and Analysis Platform (TCMSP) (https://tcmspw.com/tcmsp.php). The potential miRNA targets of LPS-binding protein were predicted via the miRwalk (http://mirwalk.umm.uni-heidelberg.de/), Targetscan (http://www.targetscan.org/mamm_31/) and miRDB (http://mirdb.org/) database.

### Statistical analysis

The SPSS version 21.0 software was used for data analyses. Statistical significance was confirmed using a Student’s *t* test. Statistically significant differences were defined as p < 0.05.

## Results

### PF inhibits the cell function and inflammation of THP-1 cells induced by LPS

**Fig. 1:**
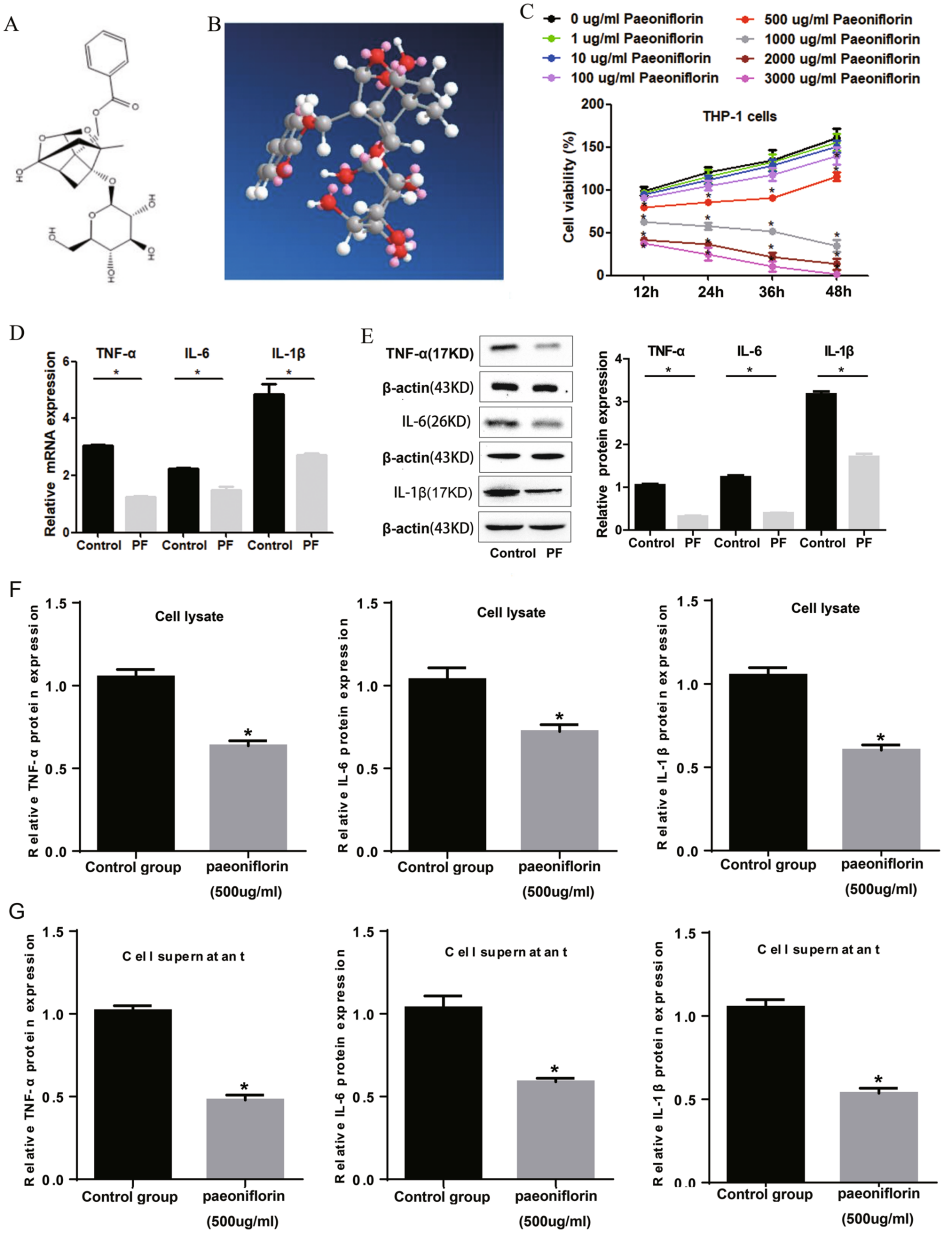
PF inhibits the proliferation and the release of pro-inflammatory cytokines of THP-1 cells. The 2D (**a**) and 3D (**b**) structures of PF. **c** 500 ug/ml PF significantly inhibited the proliferation of THP-1 cells. ELISA results showed that PF inhibited the protein expression of IL-6, IL-1β and TNF-α in the cell lysate (**d**) and supernatant (**e**) of THP-1 cells. *Represents *p* < 0.05, *NS* represents no significant difference

The molecular formula of PF (PubChem ID: 442,534) is C_23_H_28_O_11_, with molecular weight: 480.5 g/mol. As provided by PubChem database, the 2D and 3D structure of PF were shown in Fig. 1a and b. Through CCK-8 detection, we found that 500–3000 ug/ml PF significantly inhibited the cell viability of THP-1 cells (Fig. 1c). Since M2 type macrophages help to improve SLE (Li et al. 2016), 1000–3000 ug/ml PF seems to be over-dose for macrophages. Therefore, we choose 500 ug/ml PF for follow-up experiments. Through ELISA experiments, we found that 500 ug/ml PF inhibited the expression of TNF-α, IL-6 and IL-1βin the cell lysate (Fig. 1d) and cell supernatant (Fig. 1e) of THP-1 cells. These results suggest that 500ug/mL PF can not only substantially inhibit the proliferation of THP-1 cells, but also substantially reduce the secretion of TNF-α, IL-6 and IL-1βin THP-1 cells. It is suggested that 500 ug/mL PF may reduce the secretion of TNF-α, IL-6 and IL-1β by inhibiting the proliferation of THP-1 cells.

**Fig. 2:**
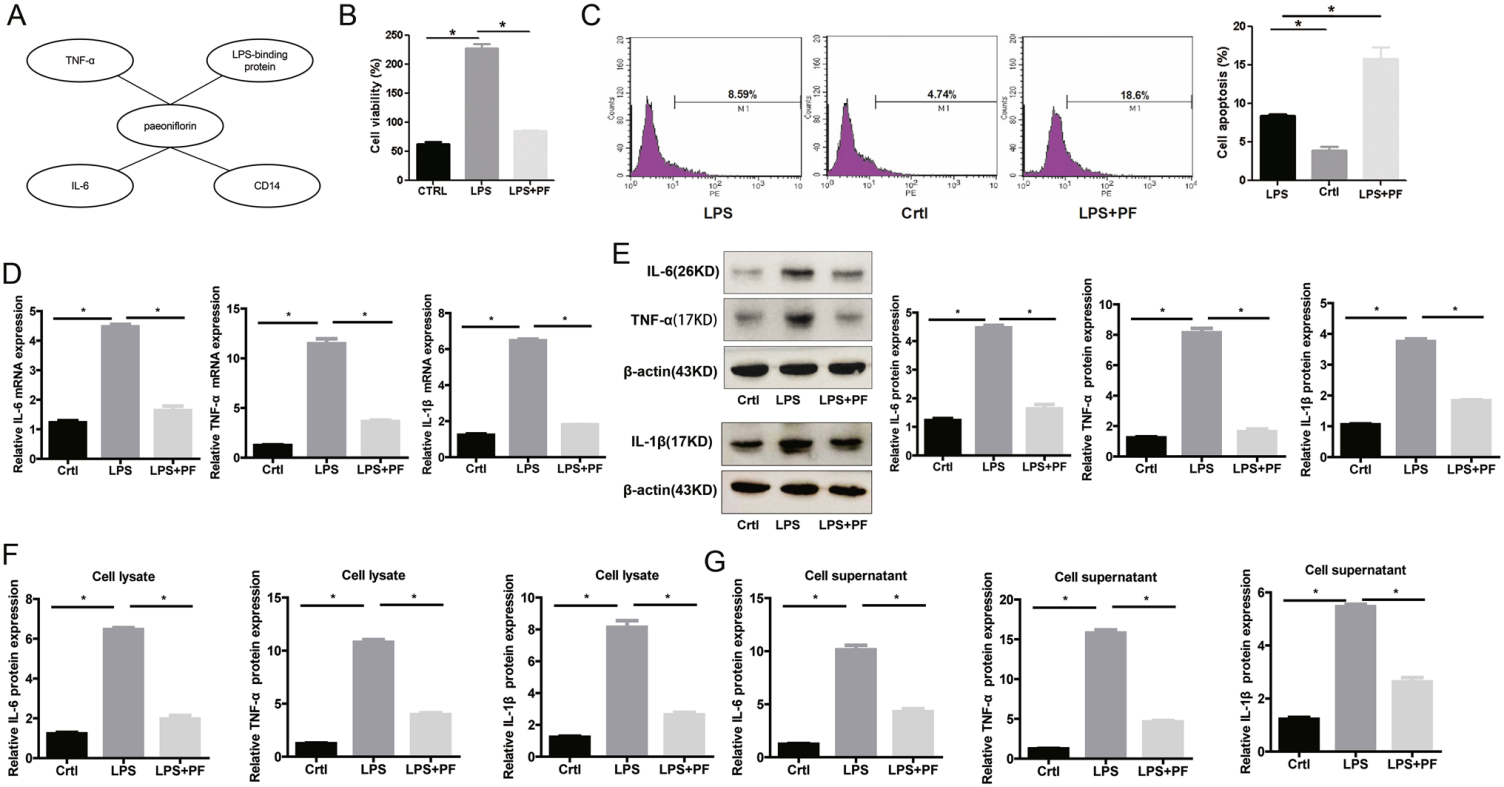
500 ug/ml PF inhibits the activity and inflammation of THP-1 cells induced by LPS. **a **Bioinformatics prediction of targets of PF. **b** PF inhibits increased cell viability of THP-1 cells induced by LPS. **c** PF promotes the apoptosis of THP-1 cells. PF inhibits the increase in mRNA (**d**) and protein (**e**–**g**) expression of IL-6, IL-1β and TNF-α induced by LPS via RT-PCR, WB and ELISA detection. *Represents p < 0.05; CTRL represents control group; β-actin and GAPDH are used as an internal control

Through bioinformatics prediction via the TCMSP database, we found that PF might be involved in the regulation of TNF-α, IL-6, CD14 and LPS-binding protein (Fig. 2a). CCK-8 and flow cytometry detection showed that 500 ug/ml PF could significantly inhibit the cell viability (Fig. 2b) while promote the apoptosis (Fig. 2c) of THP-1 cells. In addition, the increase in mRNA (Fig. 2d) and protein (Fig. 2e–g) expression of TNF-α, IL-6 and IL-1β induced by LPS could be significantly reversed by 500 ug/ml PF. Together, 500ug/ml PF could inhibit the pro-inflammatory response induced by LPS.

### LPS regulates the cell function and inflammation of THP-1 cells by inhibiting the expression of miR-124

**Fig. 3:**
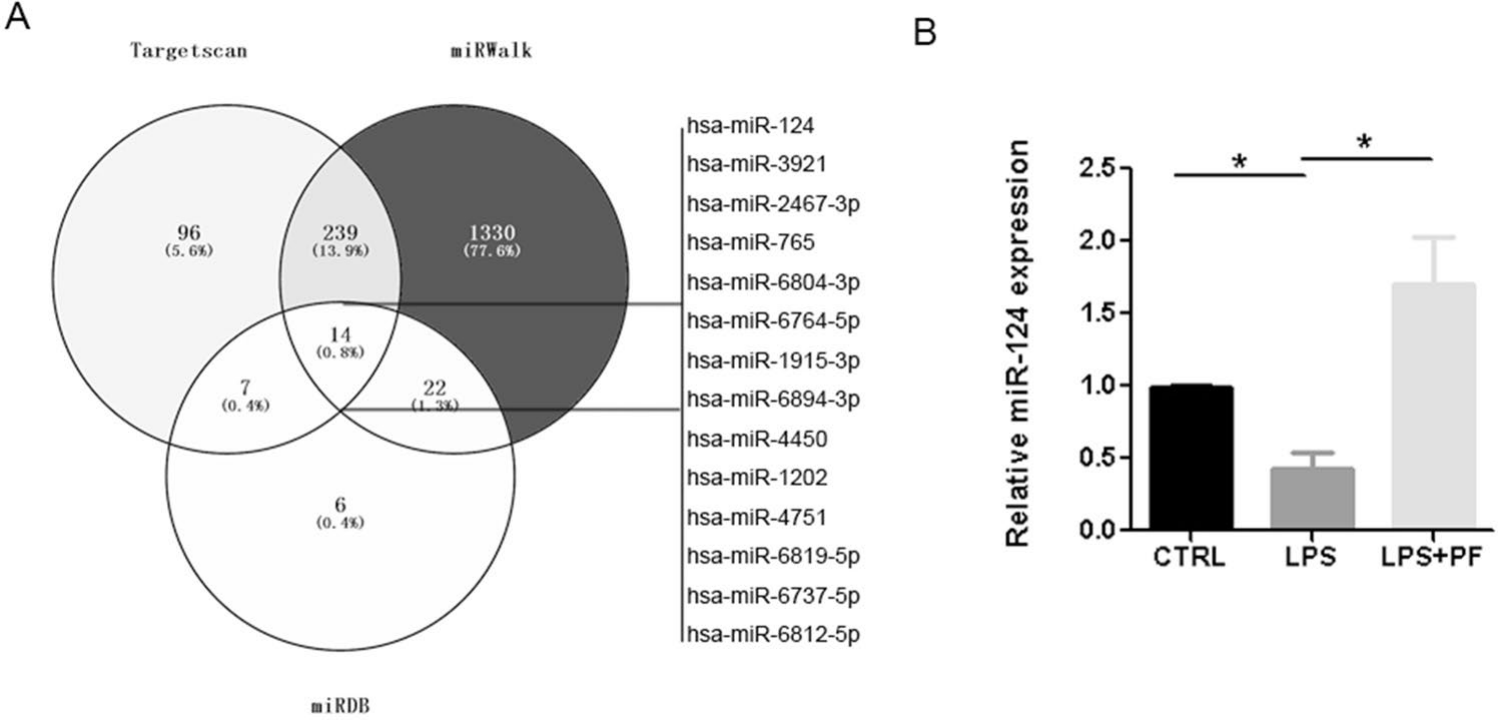
PF up-regulates the expression of miR-124 in THP-1 cells. **a** Prediction of miRNA target of LPS-binding protein. **b** The down-regulated expression of miR-124 induced by LPS was significantly reversed by 500 ug/ml PF. *Represents p < 0.05; U6 is used as an internal control

**Fig. 4:**
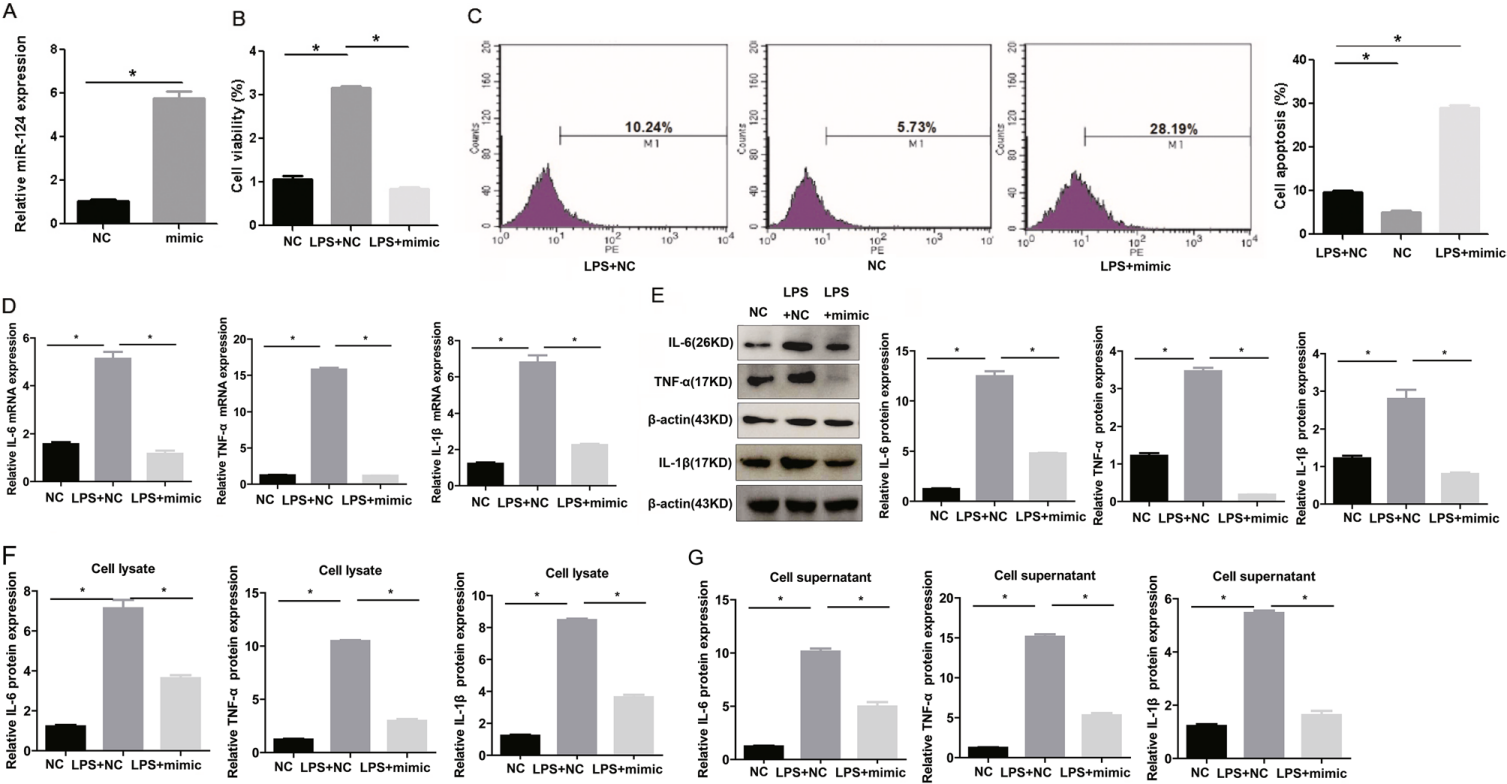
LPS regulates the biological function and inflammation of THP-1 cells depending on the down-regulated expression of miR-124. **a** The efficiency of miR-124-mimic was tested by RT-PCR. Compared with the LPS + mimic-NC group, the proliferation of THP-1 cells in the LPS + miR-124-mimic group decreased significantly (**b**), while the apoptosis increased significantly (**c**). Compared with the LPS + mimic-NC group, the mRNA (**d**) and protein (**e–g**) expression of IL-6, IL-1β and TNF-α in the LPS + miR-124-mimic group were significantly reduced via RT-PCR, WB and ELISA detection. *Represents p < 0.05; U6, β-actin and GAPDH are used as an internal control

Through the bioinformatics prediction via the miRwalk, Targetscan and miRDB database, we found that there are 14 potential miRNA targets for LPS-binding protein **(**Fig. 3a). Therefore, we speculate that LPS might be involved in regulating the expression of these miRNAs. RT-PCR results showed that LPS inhibited the expression of miR-124. However, 500 ug/ml PF significantly reversed the down-regulation of miR-124 induced by LPS (Fig. 3b). RT-PCR results showed that the expression of miR-124 in the miR-124-mimic group was significantly increased by about 6 times compared with the mimic-NC group **(**Fig. 4a**)**. And overexpression of miR-124 significantly inhibited the increase in cell viability induced by LPS by CCK-8 detection **(**Fig. 4b**)**. The results of flow cytometry showed that overexpression of miR-124 significantly promoted the apoptosis of THP-1 cells **(**Fig. 4c**)**. In addition, overexpression of miR-124 could significantly reverse the increase of TNF-α, IL-6 and IL-1β expression in mRNA (Fig. 4d) and protein (Fig. 4e–g) levels induced by LPS. Together, PF regulates the biological function and inflammation of THP-1 cells by down-regulating the expression of miR-124.

### 500 ug/ml PF up‐regulates miR-124 to regulate the biological functions induced by LPS in THP-1 cells

**Fig. 5:**
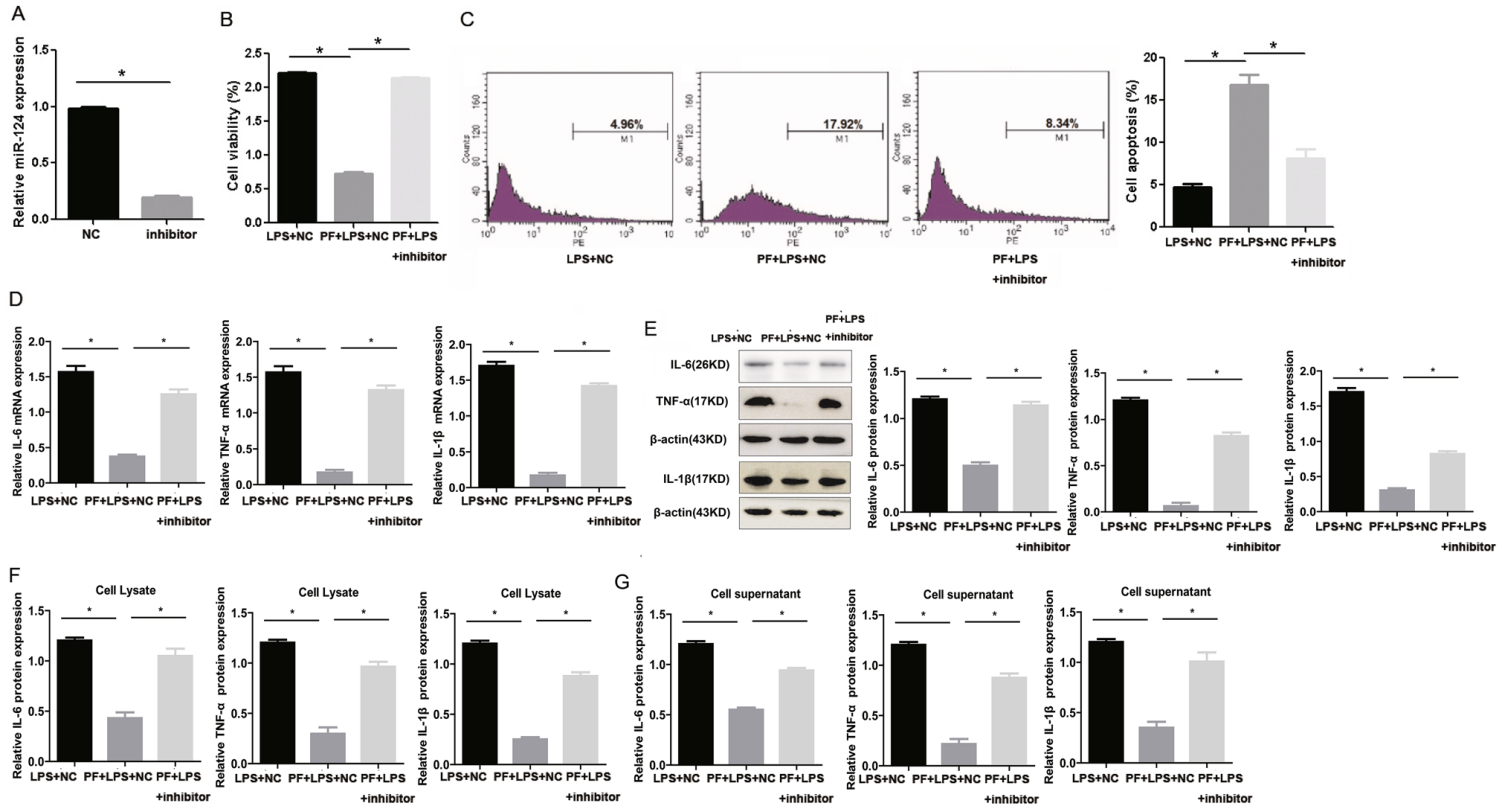
500 ug/ml PF significantly reversed the cell function and inflammation induced by LPS in THP-1 cells. **a** The efficiency of miR-124-inhibitor was tested by RT-PCR. Compared with the PF + LPS + inhibitor-NC group, the proliferation of THP-1 cells in the PF + LPS + miR-124-inhibitor group increased significantly (**b**), while the apoptosis decreased significantly (**c**). Compared with the PF + LPS + inhibitor-NC group, the mRNA (**d**) and protein (**e–g**) expression of IL-6, IL-1β and TNF-α in the PF + LPS + miR-124-inhibitor group were significantly increased via RT-PCR, WB and ELISA detection. *Represents p < 0.05; U6, β-actin and GAPDH are used as an internal control

As shown in Fig. 3a, PF significantly promoted the expression of miR-124 in THP-1 cells. RT-PCR results showed that compared with the inhibitor-NC group, the expression of miR-124 was significantly reduced in the miR-124-inhibitor group (Fig. 5a). CCK-8 results showed that compared with the PF + LPS + inhibitor-NC group, the cell viability of THP-1 cells in the PF + LPS + miR-124-inhibitor group was significantly increased (Fig. 5b). The results of flow cytometry showed that compared with the PF + LPS + inhibitor-NC group, in the PF + LPS + miR-124-inhibitor group, the apoptosis of THP-1 cells was significantly reduced (Fig. 5c). In addition, compared with the PF + LPS + inhibitor-NC group, the mRNA (Fig. 5d) and protein (Fig. 5e–g) expression of TNF-α, IL-6 and IL-1β in the PF + LPS + miR-124-inhibitor group increased significantly. Together, 500ug/ml PF inhibits the activity and inflammation of THP-1 cells by up-regulating the expression of miR-124.

## Discussion

In this work, we characterized the biological role of PF in THP-1 cells, a macrophage suppressor that inhibits cell proliferation and promotes cell apoptosis by up-regulating the expression of miR-124. In addition, PF inhibits the expression of TNF-α, IL-6 and IL-1β by up-regulating miR-124.

PF has been found to inhibit tumor growth in a variety of tumors (Zhang et al. 2018). However, whether PF inhibits the growth of macrophages remained unknown. In the present study, we found for the first time that 500 ug/ml PF could significantly inhibit the proliferation of THP-1 cells, but 1, 10 and 100 ug/ml PF have no significant effect on the biological function of THP-1 cells. In addition, M2 type macrophages is beneficial for improving SLE (Li et al. 2016) and thus 1000–3000 ug/ml PF seems to be over-dose for macrophages. Therefore, 500 ug/ml PF were used in the subsequent experiments to further explore the biological function of PF in THP-1 cells.

LPS has the function of promoting cell proliferation and inflammation (Fritsche 2015). In this study, we found that LPS promotes the proliferation and the apoptosis of THP-1 cells. In addition, LPS can significantly promote the release of IL-6, IL-1β and TNF-α in THP-1 cells. However, 500ug/ml PF significantly reversed the pro-proliferation and pro-inflammatory effects induced by LPS in THP-1 cells. Therefore, PF may reduce the release of IL-6, IL-1β and TNF-α by inhibiting the proliferation of THP-1 cells.

IL-1β and TNF-α have strong pro-inflammatory activity and can promote the secretion of a variety of pro-inflammatory mediators, including IL-6, IL-8 and IL-l0 (Wang et al. 2020). Zhu et al. (2018)reported that compared with healthy controls, IL-6 was elevated in the plasma of leukemia patients, and IL-6 supported the survival of leukemia cells in vivo/in vitro. Parnsamut and Brimson (2015) reported that gold nanoparticles inhibited the cell proliferation of monocytic U937 cells by down-regulating the expression of TNF-α. These results suggest that the inhibition of IL-6, IL-1β and TNF-α by PF may further reduce the proliferation of THP-1 cells.

Abnormal expression of miR-124 has been found in many inflammatory and immune diseases, and it often acts as a negative regulator of inflammation or immune signals (Qin et al. 2016). In our study, 500ug/ml PF was found significantly up-regulated the expression of miR-124 in THP-1 cells. Further function gain-and-loss experiments indicated that the increase in cell viability and the release of IL-6, IL-1β and TNF-α induced by 100 ng/mL LPS were significantly reversed by the miR-124. As reported before, miR-124 inhibits the proliferation of macrophages (Zhai et al. 2020), and inhibits the expression of TNF-α, IL-6 and IL-1β (He et al. 2019; Ohnuma et al. 2019). In addition, miR-124 is essential for inducing and maintaining the M2 phenotype of macrophages (Veremeyko et al. 2013). Therefore, PF may inhibit the proliferation of THP-1 cells and the release of IL-6, IL-1β and TNF-α by up-regulating miR-124. However, whether PF directly inhibits the release of IL-6, IL-1β and TNF-α in THP-1 cells by up-regulating miR-124, in a way that does not depend on the inhibition of proliferation, needs further verification.

**Fig. 6:**
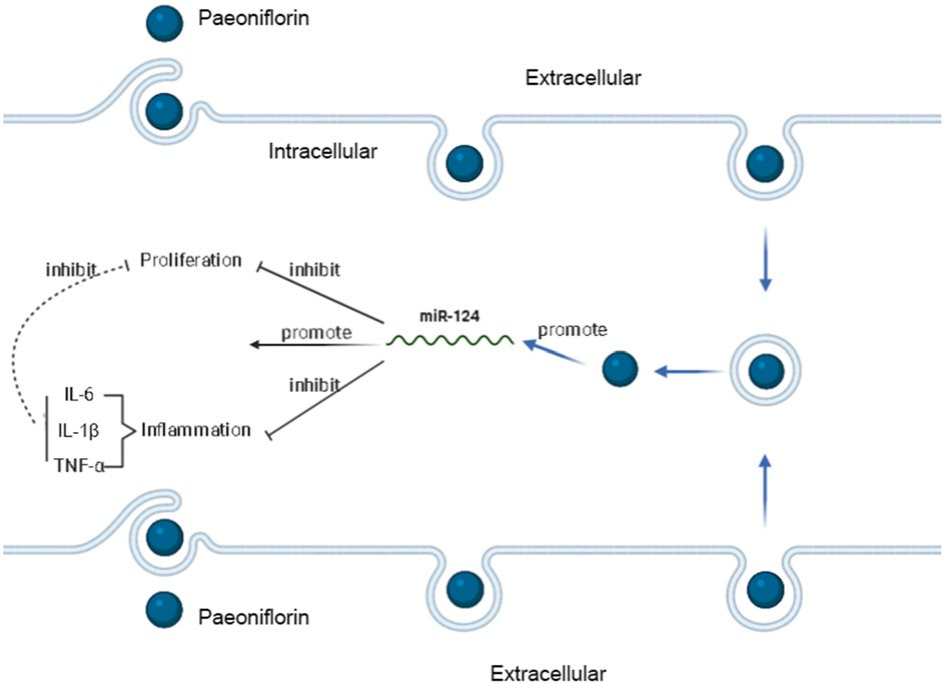
Schematic diagram of PF and the biological functions of THP-1 cells

In conclusion, this study initially elucidates that PF inhibits the proliferation and the release of proinflammatory cytokines of THP-1 cells by promoting the expression of miR-124 (Fig. 6). And, our preliminary results support the clinical potential of PF for the treatment of SLE patients.

## References

[CR1] Chanput W, Mes JJ, Savelkoul HF, Wichers HJ (2013). Characterization of polarized THP-1 macrophages and polarizing ability of LPS and food compounds. Food Funct.

[CR2] Fan X, Wu J, Yang H, Yan L, Wang S (2018). Paeoniflorin blocks the proliferation of vascular smooth muscle cells induced by plateletderived growth factorBB through ROS mediated ERK1/2 and p38 signaling pathways. Mol Med Rep.

[CR3] Fan HN, Liao XH, Zhang J, Zheng HM (2020). Macrophages promote cell proliferation in colorectal cancer via IL-1beta-mediated downregulation of miR-28-3p. J Biol Regul Homeost Agents.

[CR4] Fritsche KL (2015). The science of fatty acids and inflammation. Adv Nutr.

[CR5] Funes SC, Rios M, Escobar-Vera J, Kalergis AM (2018). Implications of macrophage polarization in autoimmunity. Immunology.

[CR6] He F, Zhang C, Huang Q (2019). Long noncoding RNA nuclear enriched abundant transcript 1/miRNA-124 axis correlates with increased disease risk, elevated inflammation, deteriorative disease condition, and predicts decreased survival of sepsis. Medicine.

[CR7] Kiriakidou M, Ching CL (2020). Systemic lupus erythematosus. Ann Intern Med.

[CR8] Li F, Zhu X, Yang Y, Huang L, Xu J (2016). TIPE2 alleviates systemic lupus erythematosus through regulating macrophage polarization. Cell Physiol Biochem.

[CR9] Liu B, Lin J, Bai L, Zhou Y, Lu R, Zhang P, Chen D, Li H, Song J, Liu X (2019). Paeoniflorin inhibits mesangial cell proliferation and inflammatory response in rats with mesangial proliferative glomerulonephritis through PI3K/AKT/GSK-3beta pathway. Front Pharmacol.

[CR10] Ma C, Xia Y, Yang Q, Zhao Y (2019). The contribution of macrophages to systemic lupus erythematosus. Clin Immunol.

[CR11] Meng Y, Beckett MA, Liang H, Mauceri HJ, van Rooijen N, Cohen KS, Weichselbaum RR (2010). Blockade of tumor necrosis factor alpha signaling in tumor-associated macrophages as a radiosensitizing strategy. Cancer Res.

[CR12] Ohnuma K, Kasagi S, Uto K, Noguchi Y, Nakamachi Y, Saegusa J, Kawano S (2019). MicroRNA-124 inhibits TNF-alpha- and IL-6-induced osteoclastogenesis. Rheumatol Int.

[CR13] Parnsamut C, Brimson S (2015). Effects of silver nanoparticles and gold nanoparticles on IL-2, IL-6, and TNF-alpha production via MAPK pathway in leukemic cell lines. Genet Mol Res.

[CR14] Qin Z, Wang PY, Su DF, Liu X (2016). miRNA-124 in immune system and immune disorders. Front Immunol.

[CR15] Veremeyko T, Siddiqui S, Sotnikov I, Yung A, Ponomarev ED (2013). IL-4/IL-13-dependent and independent expression of miR-124 and its contribution to M2 phenotype of monocytic cells in normal conditions and during allergic inflammation. PLoS One.

[CR16] Wang QM, Wang H, Li YF, Xie ZY, Ma Y, Yan JJ, Gao YF, Wang ZM, Wang LS (2016). Inhibition of EMMPRIN and MMP-9 expression by epigallocatechin-3-gallate through 67-kDa laminin receptor in PMA-induced macrophages. Cell Physiol Biochem.

[CR17] Wang Y, Che M, Xin J, Zheng Z, Li J, Zhang S (2020). The role of IL-1beta and TNF-alpha in intervertebral disc degeneration. Biomed Pharmacother.

[CR18] Yao J, Du X, Chen S, Shao Y, Deng K, Jiang M, Liu J, Shen Z, Chen X, Feng G (2018). Rv2346c enhances mycobacterial survival within macrophages by inhibiting TNF-alpha and IL-6 production via the p38/miRNA/NF-kappaB pathway. Emerg Microbes Infect.

[CR19] Zhai C, Cong H, Hou K, Hu Y, Zhang J, Zhang Y, Zhang Y, Zhang H (2020). Effects of miR-124-3p regulation of the p38MAPK signaling pathway via MEKK3 on apoptosis and proliferation of macrophages in mice with coronary atherosclerosis. Adv Clin Exp Med.

[CR20] Zhang J, Li H, Huo R, Zhai T, Li H, Sun Y, Shen B, Li N (2015). Paeoniflorin selectively inhibits LPS-provoked B cell function. J Pharmacol Sci.

[CR21] Zhang J, Yu K, Han X, Zhen L, Liu M, Zhang X, Ren Y, Shi J (2018). Paeoniflorin influences breast cancer cell proliferation and invasion via inhibition of the Notch1 signaling pathway. Mol Med Rep.

[CR22] Zhang CX, Wang HY, Yin L, Mao YY, Zhou W (2020). Immunometabolism in the pathogenesis of systemic lupus erythematosus. J Transl Autoimmun.

[CR23] Zhu F, McCaw L, Spaner DE, Gorczynski RM (2018). Targeting the IL-17/IL-6 axis can alter growth of chronic lymphocytic leukemia in vivo/in vitro. Leuk Res.

